# Supplementary material for: Rapid modeling of experimental molecular kinetics with simple electronic circuits instead of with complex differential equations
Source: Front Bioeng Biotechnol. 2022 Sep 28;10:947508. doi: 10.3389/fbioe.2022.947508 (PMC9554301; doi:10.3389/fbioe.2022.947508)
Supplement: Supplementary file 3 [file DataSheet1.pdf]

Supplementary Material

Rapid modeling of experimental molecular kinetics with simple electronic circuits instead of with complex differential equations

Yijie Deng[1], Douglas Raymond Beahm[1], Xinping Ran[1], Tanner G Riley[2], Rahul Sarpeshkar[1,3*]

1. Thayer School of Engineering, Dartmouth College, Hanover, NH, 03755, USA
2. School of Undergraduate Arts and Sciences, Dartmouth College, Hanover, NH, 03755, USA
3. Departments of Engineering, Microbiology and Immunology, Physics, and Molecular and Systems Biology, Dartmouth College, Hanover, NH, 03755, USA

* Correspondence: rahul.sarpeshkar@dartmouth.edu

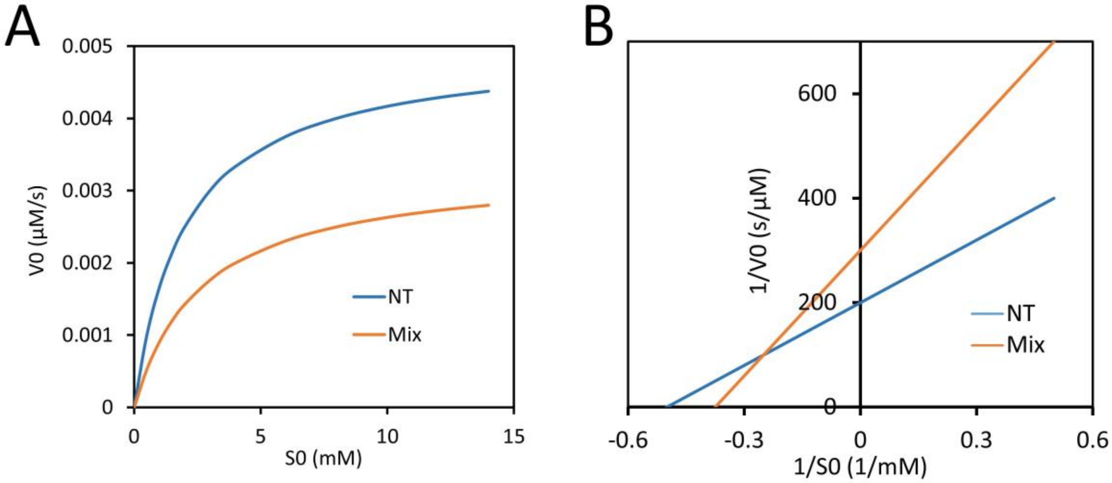

Figure S1. Circuit modeling of enzyme inhibition by a mixed-type inhibitor. **(A)** Model-predicted curves for initial reaction rate (V_0) versus initial substrate concentration (S_0) with and without inhibitor. **(B)** Lineweaver-Burk plot for $1/V_0$ versus $1/S_0$ curves, with and without inhibitor, predicted by the circuit model. NT: reactions without inhibitor; Mix: reactions with mixed inhibition. The model curves are predicted by the circuit in Figure 8A. For comparison, all parameters used in this figure were the same as in those Figure 8, including K_m = 2 mM, K_i = 3 mM, k_{cat} = 500/s, E_0 = 10 nM, and inhibitor I_0 = 3 mM. The extra parameters used for the mixed-inhibition, K_{i2} = 6 mM and K_{m2} = 4 mM, are different from K_i and K_m, respectively.

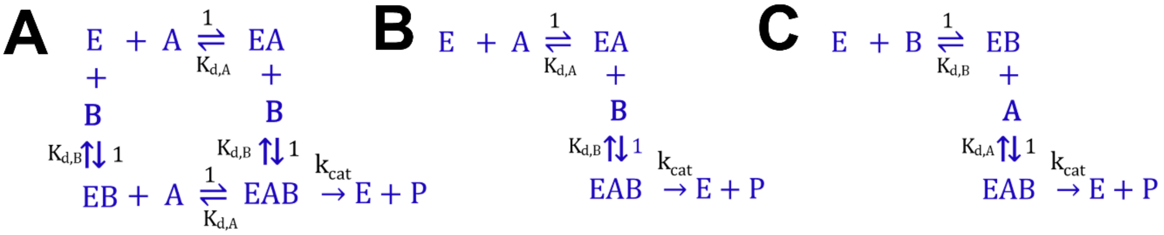

D

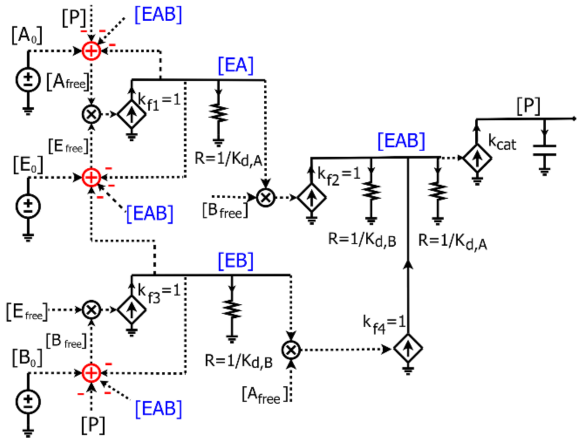

E

Reactions	Parameter setting
Random binding Reaction (A)	By default
EA+B Reaction (B)	k_{f3} = k_{f4} = 0, with the resistor (1/$K_{d,A}$) removed
EB+A Reaction (C)	k_{f1} = k_{f2} = 0, with the resistor (1/$K_{d,B}$) removed

Figure S2 Circuit modeling of an enzymatic reaction with two substrates. **(A)** A generalized reaction schematic for two substrates randomly binding to enzyme E, forming a triad complex to generate the product P with a rate constant of k_{cat}. This reaction has four binding parameters and one rate constant for the catalytic reaction. **(B)** Ordered-binding reaction scheme (EA+B) with the enzyme first binding to substrate A and then to B. **(C)** Ordered-binding reaction scheme (EB+A) with the enzyme first binding to substrate B and then to A. **(D)** Circuit model of the generalized reaction with two substrates. This circuit is similar to the one in Figure 7D. Three modifications are made: (1) ESI binding is replaced by the bindings of substrate A and B to enzyme; (2) the catalytic reaction is from the [EAB] complex; (3) P needs to be subtracted from both substrates A and B, with the "use-it-and-lose-it" mass conservation rule. The circuit was directly translated from the reaction schematic in (A) without deriving any mathematic equations. **(E)** Circuit parameter settings for the different reaction schemes. Using these parameter settings, this generalized circuit can also model the ordered-binding reactions in (**B**) and (**C**).

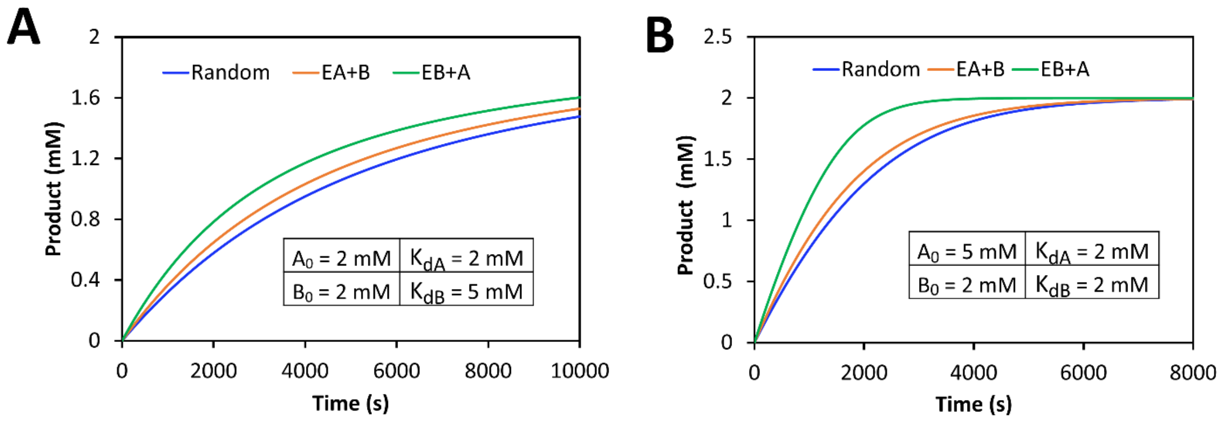

Figure S3 Circuit simulation of product dynamics for two-substrate reactions with different binding orders. **(A)** Reactions with the same concentration for both substrates but different K_d values. **(B)** Reactions with the same K_d value for both substrates but different substrate concentrations. Random: random-binding reaction; EA+B: enzyme binds to A then B; EB+A: enzyme binds to B then A. The parameters used in the simulations are listed in the inserted table in each graph. The two common parameters used are: E_0 = 10 nM, k_{cat} = 500/s.

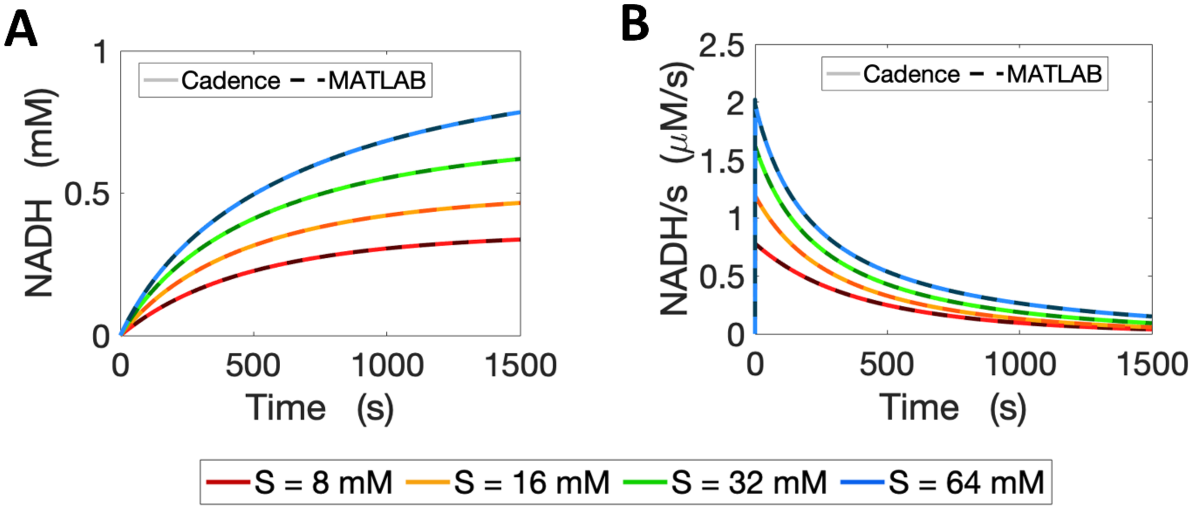

Figure S4 Comparison of MATLAB and Cadence simulation results. **(A)** Using the yeast ADH reversible reaction circuit model from Figure 11, we compared the solutions from MATLAB (dashed lines, dark colors) and Cadence (solid lines, light colors) simulations of product (NADH) formation dynamics for different initial amounts of substrate (S_0). The results were virtually identical. **(B)** The net NADH production rate (NADH/s) has a near-vertical line just after zero seconds for both simulators. The steep line is a result of the alcohol substrate being added at time zero, allowing for the rapid formation of downstream intermediate complexes that quickly increase the NADH production rate from its initial rate of zero to its peak rate. The MATLAB simulation was performed by the stiff ODE solver "ode15s." The Cadence simulation was performed using the built-in ADE L simulation window, which runs Cadence's SPICE-based circuit simulator called Spectre.

Table S1. Rate constants used in modeling the reversible reaction by yeast ADH.

Rate constants	Values	Cited values	Units	References
k_1	$2x10^6$	$2x10^6$	$M^{-1}s^{-1}$	(1, 2)
k_{-1}	$1.9x10^3$	$1.9x10^3$	s^{-1}	
k_2	$59x10^3$	$59x10^6$	$M^{-1}s^{-1}$	
k_{-2}	$1x10^3$	$1x10^3$	s^{-1}	
k_3	$8x10^3$	$4x10^3$	s^{-1}	
k_{-3}	$35x10^3$	$35x10^3$	s^{-1}	
k_4	$22x10^3$	$11x10^3$	s^{-1}	
k_{-4}	$4.3x10^6$	$4.3x10^6$	$M^{-1}s^{-1}$	
k_5	960	480	s^{-1}	
k_{-5}	$15x10^6$	$15x10^6$	$M^{-1}s^{-1}$	

Note: All parameters used in our model are identical or near to values cited in our references except k_2, which needed a 1000-fold decrease to give us a good fit to measured experimental data and to yield a K_m for ethanol binding, which is consistent with other measured values (2). The k_2 reported in (1), likely an overestimate, leads to a smaller than usual K_m for ethanol binding and saturates the reactions at all ethanol concentrations used in our study.

Table S2 Kinetic parameters used for TXTL in the *E.coli*-based cell-free system

Parameters	Notation	Values used [a, b]	Parameters in circuits [a, b]	Reference values	References
K_m_T7	Dissociation constant for T7 RNAP and the T7 promoter	4.8 nM	R = 1/K_m = 2.08*10^8 Ω	4.8 nM	(3)
[$RNAP_0$]	T7 RNAP concentration used	16 nM	16 nV	This study	
Lm	Total mRNA length	900 nt	900 nt	This study (DA313 plasmid)	
Lp	GFP coding region length	717 nt	717 nt		
Cm	T7 RNAP speed	50 nt/s	50 nt/s	43 nt/s	(4)
k_{TX}	Transcription rate	0.09 1/s	0.09 A/V	0.065 1/s	(5)
k_{TL}	Translation rate	0.0064 1/s (0.0055 1/s)	0.0064 A/V (0.0055 A/V)	0.006 1/s	
Cp	Ribosome speed	1.38 nt/s (1.2 nt/s)	1.38 nt/s (1.2 nt/s)	2.5 nt/s	
k_{mat}	GFP maturation rate	0.0014 1/s	0.0014 A/V	0.000725 1/s	
N_RNAP	RNAP consumed per DNA-RNAP complex during transcription	N = 1 + K_TX * (Lm/Cm) = 2.62	k = 2.62	N/A	
N_Ribo	Ribosome consumed per mRNA-Ribo complex during translation	N = 1 + k_TL * (Lp/Cp) = 4.33 (N = 4.29)	k = 4.33 (k = 4.29)	~2.92	
[$Ribo_0$]	Total ribosome concentration	1500 nM (850 nM)	1500 nV (850 nV)	1100 nM	
K_{Ribo}	Dissociation constant for ribosome binding to RBS on mRNA	10 nM	R = 1/Km = 10^8 Ω	10 nM	
d	mRNA degradation rate	0.00091 1/s (0.00118 1/s)	R =1/d =1100 Ω (R = 850 Ω)	0.00083 1/s	
K_i_TetR	Dissociation constant for TetR dimer and tetO	5 nM	R= 1/K_i = 2*10^8 Ω	0.18-13 nM	(6, 7)

Note: In our circuits, Michaelis-Menten constants (K_m) were used interchangeably with the dissociation constants (K_d). All values are the same or comparable to the literature cited with only slight variations for different experimental conditions. **a:** The values in parentheses are parameters used in the modeling of TetR regulation with slight variations to account for different batches/preparations of cell-free lysate and plasmid. **b:** For circuits under a steady-state approximation, capacitors corresponding to binding state variables are removed such that resistors are directly related to steady-state Michaelis-Menten constants (K_m) only, and do not affect dynamic parameters like time constants.

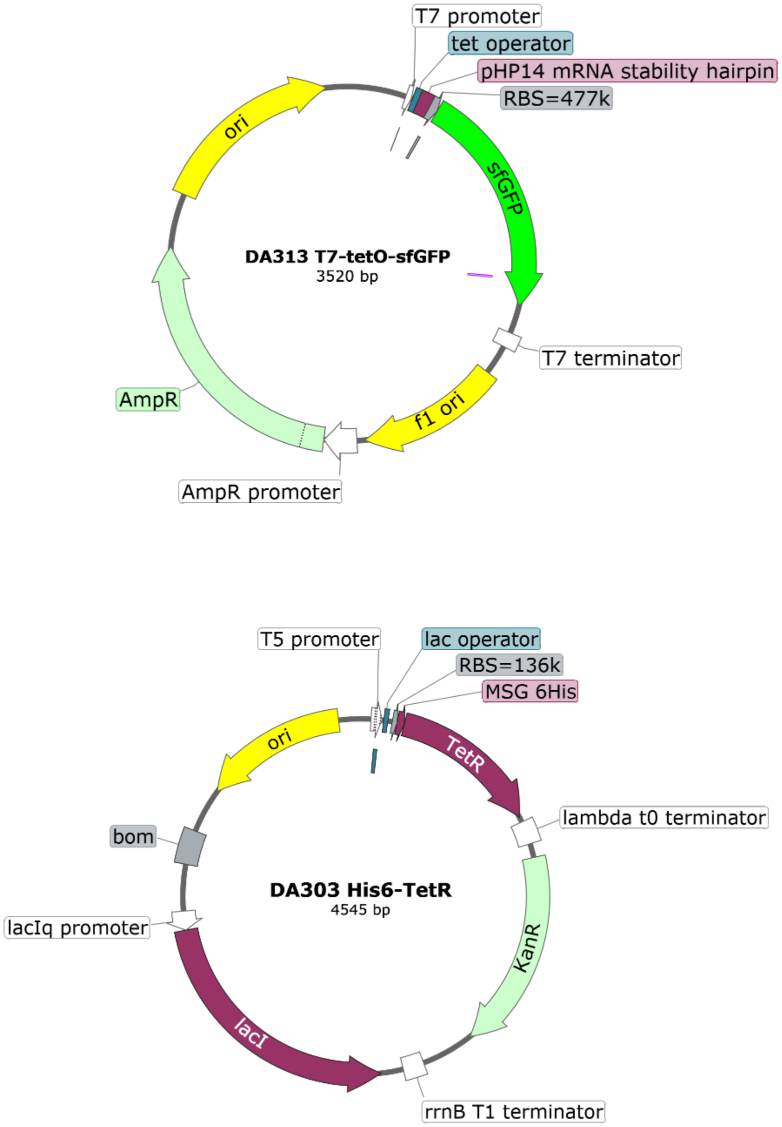

Figure S5 Maps of the plasmids constructed in this work. DA313 is the plasmid used in the cell-free system while DA303 is the plasmid for the preparation of the recombinant TetR protein.

Supplementary References

1. Dickinson FM, Dickenson CJ. 1978. Estimation of rate and dissociation constants involving ternary complexes in reactions catalysed by yeast alcohol dehydrogenase. Biochem J 171:629–37.

2. Ganzhorn AJ, Green DW, Hershey AD, Gould RM, Plapp B V. 1987. Kinetic characterization of yeast alcohol dehydrogenases. Amino acid residue 294 and substrate specificity. J Biol Chem 262:3754–3761.

3. Újvári A, Martin CT. 1996. Thermodynamic and kinetic measurements of promoter binding by T7 RNA polymerase. Biochemistry 35:14574–14582.

4. Skinner GM, Baumann CG, Quinn DM, Molloy JE, Hoggett JG. 2004. Promoter binding, initiation, and elongation by bacteriophage T7 RNA polymerase: A single-molecule view of the transcription cycle. J Biol Chem 279:3239–3244.

5. Marshall R, Noireaux V. 2019. Quantitative modeling of transcription and translation of an all-E. coli cell-free system. Sci Rep 9:11980.

6. Kamionka A, Bogdanska-Urbaniak J, Scholz O, Hillen W. 2004. Two mutations in the tetracycline repressor change the inducer anhydrotetracycline to a corepressor. Nucleic Acids Res 32:842–847.

7. Kędracka-Krok S, Wasylewski Z. 1999. Kinetics and equilibrium studies of Tet repressor-operator interaction. J Protein Chem 18:117–125.
