# Supplementary figures and images for: Rapid modeling of experimental molecular kinetics with simple electronic circuits instead of with complex differential equations
Source: Front Bioeng Biotechnol. 2022 Sep 28;10:947508. doi: 10.3389/fbioe.2022.947508 (PMC9554301; doi:10.3389/fbioe.2022.947508)

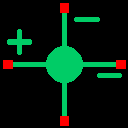

Supplement: Supplementary file 4 [file DataSheet2.ZIP › MolecularKinetics_Deng2022/subtractor_1p2n/symbol/thumbnail_128x128.png]

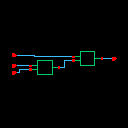

Supplement: Supplementary file 4 [file DataSheet2.ZIP › MolecularKinetics_Deng2022/subtractor_1p2n/schematic/thumbnail_128x128.png]

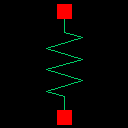

Supplement: Supplementary file 4 [file DataSheet2.ZIP › MolecularKinetics_Deng2022/res/auCdl/thumbnail_128x128.png]

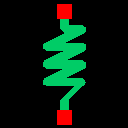

Supplement: Supplementary file 4 [file DataSheet2.ZIP › MolecularKinetics_Deng2022/res/symbol/thumbnail_128x128.png]

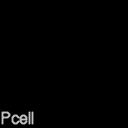

Supplement: Supplementary file 4 [file DataSheet2.ZIP › MolecularKinetics_Deng2022/res/symbol_xform/thumbnail_128x128.png]

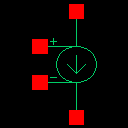

Supplement: Supplementary file 4 [file DataSheet2.ZIP › MolecularKinetics_Deng2022/vccs/auCdl/thumbnail_128x128.png]

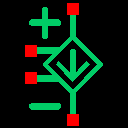

Supplement: Supplementary file 4 [file DataSheet2.ZIP › MolecularKinetics_Deng2022/vccs/symbol/thumbnail_128x128.png]

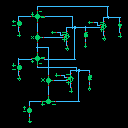

Supplement: Supplementary file 4 [file DataSheet2.ZIP › MolecularKinetics_Deng2022/Fig5_Competitive_Inhibition/schematic/thumbnail_128x128.png]

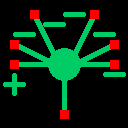

Supplement: Supplementary file 4 [file DataSheet2.ZIP › MolecularKinetics_Deng2022/subtractor_1p5n/symbol/thumbnail_128x128.png]

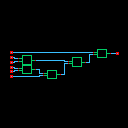

Supplement: Supplementary file 4 [file DataSheet2.ZIP › MolecularKinetics_Deng2022/subtractor_1p5n/schematic/thumbnail_128x128.png]

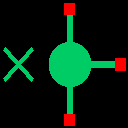

Supplement: Supplementary file 4 [file DataSheet2.ZIP › MolecularKinetics_Deng2022/multiplier/symbol/thumbnail_128x128.png]

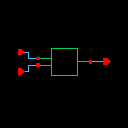

Supplement: Supplementary file 4 [file DataSheet2.ZIP › MolecularKinetics_Deng2022/multiplier/schematic/thumbnail_128x128.png]

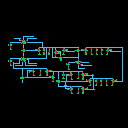

Supplement: Supplementary file 4 [file DataSheet2.ZIP › MolecularKinetics_Deng2022/Fig11andS4_Reversible_Reaction_Yeast_ADH/schematic/thumbnail_128x128.png]

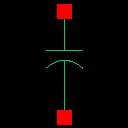

Supplement: Supplementary file 4 [file DataSheet2.ZIP › MolecularKinetics_Deng2022/cap/auCdl/thumbnail_128x128.png]

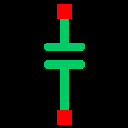

Supplement: Supplementary file 4 [file DataSheet2.ZIP › MolecularKinetics_Deng2022/cap/symbol/thumbnail_128x128.png]
